# Did the Poor Get Poorer? The Impact of COVID-19 on Social Inequalities Between International and Domestic Students

**DOI:** 10.1177/10283153221150116

**Published:** 2023-01-11

**Authors:** Anna Marczuk, Markus Lörz

**Affiliations:** 1 Research Group on Higher Education, 26567 University of Konstanz, Konstanz, Germany; 2 Educational Governance, Leibniz Institute for Research and Information in Education (DIPF), Frankfurt am Main, Germany

**Keywords:** COVID-19, international students, social inequality, higher education, study duration

## Abstract

This paper examines the influence of COVID-19 on social inequality in higher education. In particular, we focus on the study duration of international students compared to domestic ones in Germany. We assume that the pandemic has increased or decreased existing differences between both groups, affecting their study delay. The multilevel analyses with data “Studying in Germany in Corona Times” (2020) confirm most of our theoretical assumptions: on the one hand, international students expect a longer study duration due to their worsening financial situation. On the other hand, domestic students expect a longer study duration due to greater learning issues, which are provoked by a lower level of interaction in online teaching. Our results propose a more differentiated picture of the impact of the COVID-19 pandemic on social inequality: not only do financially poor international students get poorer but educationally advantaged domestic students lose, too.

## Introduction

2020 is clearly a year we will remember. The sudden outbreak of the COVID-19 pandemic has changed several life spaces, including higher education. Universities throughout the world have had to shift from in-person to online teaching and home study (Ali, 2020; Crawford et al., 2020). These changes provoked challenges for university staff members and especially for students. The rapid introduction of digital teaching has led to new *learning challenges* (Aguilera-Hermida, 2020; Gamage et al., 2020; Mseleku, 2020) and has also increased *social isolation* among university students (Debowska et al., 2020; Hamza et al., 2021; Raza et al., 2021). Furthermore, stringent contact restrictions have led to a precarious labour market situation and have increased the *financial burdens* of many university students (Ochnik et al., 2021; Rodríguez-Planas, 2022).

Some studies indicate that these aspects have increased social inequality among university students. Accordingly, the pandemic negatively affected vulnerable student groups, such as students from lower social backgrounds (Ohlbrecht & Jellen, 2021; Raaper & Brown, 2020), with disabilities (Liu, 2021), and of marginalized genders (Hunt et al., 2021; Rodríguez-Larrad et al., 2021). However, less is known about the situation of another vulnerable group—international students. These students leave their country of origin and move abroad for the purpose of study (UNESCO, 2015). Due to this change in educational context, they traditionally face higher assimilation and learning difficulties, as well as financial problems more often than domestic students (Cho & Yu, 2015; Hastings et al., 2021; Rienties et al., 2012; Whatley, 2017; Zhou & Zhang, 2014). Learning contexts and financial security are important factors related to academic success. Although these factors changed during the pandemic for all students, the question arises whether the situation has worsened for international students in particular. The newest research emphasises that international students have indeed faced learning difficulties during COVID-19 with respect to online learning and digital media (Falk, 2022; Veerasamy & Ammigan, 2022). Furthermore, international students experience social isolation and loneliness, which increases their stress level (Wilczewski et al., 2021). Many studies also emphasise the financial struggles of international students (Greenland et al., 2021; Woicolesco et al., 2022) because they are often excluded from national governmental relief programs (Firang, 2020). However, these studies focus solely on international students, without addressing the situation of domestic students. Only by considering both groups at once, we can understand if social inequalities have indeed changed during the pandemic.

We thus address the following research question: has the COVID-19 pandemic increased the social inequalities between international and domestic students, and why? We focus on Germany, where teaching in the summer semester of 2020 was completely digital (Marczuk et al., 2021) and where social inequalities traditionally are strongly pronounced: while Germany attracts high proportions of international students due to low tuition fees and high educational quality (Project_Atlas, 2019), international students here experience higher dropout rates and longer durations of study than those in other countries (Kercher, 2018). We analyse the effect of COVID-19 on the study duration of both groups, an educational outcome which has received much attention during the pandemic.

## Literature Review and Theoretical Assumptions

As mentioned above, international students in Germany already faced higher dropout rates and needed more time to complete their studies before the pandemic (Heublein, 2015; Kristen, 2014). Researchers have identified several issues linked to lower educational success of international students in Germany. First, international students face greater financial burdens, since they predominantly arrive from Asian or Southern and Eastern European countries (Middendorff et al., 2017) and often receive less support from parents because their standard wages are generally lower than those in Germany. As a result, international students often need to work to support themselves (Apolinarski & Brandt, 2018), but getting a job permit is limited for non-EU students, and jobbing does not always cover the needed expenses and also leaves less time for studying (Koenings et al., 2022; Pineda, 2018). Second, international students face learning issues due to language barriers (Morris-Lange, 2017; Thies & Falk, 2021; Wisniewski & Lenhard, 2022), especially since the language level needed for admission strongly differs from the academic form of German used at universities (Pineda, 2018). These learning issues are further amplified by discursive learning methods prevalent at German universities, which are less familiar in other countries (Heublein, 2015). Third, international students face higher social isolation due to assimilation barriers and their lack of social networks abroad (Koenings et al., 2022; Rech, 2012). Fourth, they face adaptation difficulties at German universities, which offer limited guidance but expect students to be proactive in organising their own studies (Pineda, 2018). Fifth, international students struggle with orientation issues caused by organising complex residence permits and other processes specific to the German context (Pineda, 2018).

Newer research on COVID-19 emphasises that of these aspects, three in particular have changed during the pandemic: on average, university students report increased financial burdens, higher learning difficulties and increased social isolation during the pandemic (Marczuk et al. 2021). Below, we focus on these three aspects and address why they may lead to a longer study delay for international versus domestic students in Germany. Our main assumption is that the pandemic has increased or decreased existing differences with regard to the financial, learning and social situations between these groups, affecting their study duration in different ways. While previous research focuses on the experiences of international students and tends to neglect the experiences of domestic students, we examine the situation of both groups equally.

### Financial Situation

After the outbreak of the pandemic, many students had less money for living expenses due to two main reasons. On the one hand, many working students experienced either reductions in working hours and unpaid leave or simply lost their job (Becker & Lörz, 2020). On the other hand, a large number of students reported that their parents could not offer as much financial support because their household income had worsened (Becker & Lörz, 2020; Berkes et al., 2020). In terms of finances, we assume that international students have been more negatively affected by the pandemic firstly because they more often work themselves (Apolinarski & Brandt, 2018) and thus might have lost their job and secondly because their parents’ support might be even more limited since they live and work in countries that are more affected by the pandemic (WHO, 2020) and are characterised by less evolved welfare states (Hall & Soskice, 2001; Saar et al., 2008). Therefore, we argue that the pandemic might increase the existing disadvantages of international students by introducing intensified financial difficulties.

One might assume that financial burdens do not affect study duration or may even shorten it, since students might have more time for studying, especially after losing a job. However, we argue that financial burdens might prolong the study duration: according to rational choice theory, financial disadvantages increase study costs, which in turn lower the utility of educational degrees and lead to lower educational success (Erikson & Jonsson, 1996). Following this line of reasoning, we assume that financial insecurity might require higher *time costs* for job searching and higher *psychological costs* in the form of high stress levels. Both can distract from efficient study and prolong study duration. We thus argue that the pandemic might have increased existing inequalities by augmenting the financial difficulties of international students, prolonging their study duration more than for German students.

Hypothesis 1:
*International students in particular experienced a worsening financial situation during COVID-19, more greatly prolonging their study duration in comparison to German students*.

### Social Isolation

According to the academic and social integration model of Tinto (1975), students who are less integrated into the higher education context experience lower levels of support from peers or lecturers and thus struggle to complete their studies. Previous research has shown that social isolation has increased during the pandemic: the majority of students in Germany struggled as a result of limited in-person contact with fellow students or lecturers and due to the lack of useful exchange in learning groups (Marczuk et al., 2021; Traus et al., 2020). We assume that social isolation affects those students who have profited from it the most; that is, since the study success of German students before the pandemic relied more heavily on the support of fellow students and closer contact with lecturers (Koenings et al., 2022; Rech, 2012), they should miss these aspects more. International students, on the other hand, should be less affected by contact limitations because they already experience social isolation and rely less on this support system. Thus, the pandemic might decrease existing inequalities because German students are now experiencing similar levels of social isolation as international students. Since the lack of social support slows down the study process and might lead to longer study duration, the better-integrated German students should expect a longer study duration.

Hypothesis 2:
*The social isolation during COVID-19 burdens German students more, prolonging their study duration more in comparison to international students*.

### Learning Situation

The sudden introduction of online teaching provoked significant changes in students’ learning situations: while some students cherish the increased flexibility and space for individual learning methods, others report greater learning difficulties in the digital context (Marczuk et al., 2021; Traus et al., 2020). We argue that the positive aspects of online learning should affect international students more, while the negative aspects should apply more strongly to German students.

On the one hand, online teaching might compensate for the language barriers faced by international students. We know that uploaded videos have made following course content easier because students can watch them at their own tempo by pausing or rewinding the videos (Marczuk et al., 2021). This should be especially beneficial to less fluent international students and thus compensate for their language barriers. On the other hand, German students are used to a strongly discursive education system (Heublein, 2015): learning in Germany predominantly takes place through in-person interaction and less through independent memorising of knowledge. That this interactive aspect is limited in online modalities (Marczuk et al., 2021) should burden German students more. International students, however, should be less affected by this, since they have already been less involved in seminar discussions in the past.

We argue that online teaching might decrease the learning disadvantages of international students due to two main reasons: first, by compensating for language barriers experienced by international students and second, by decreasing the activity and participation of German students. These greater learning difficulties faced by German students should in turn manifest itself in a longer study duration. Following Boudon's (1974) theoretical framework of primary and secondary effects, we argue that *performance*-related difficulties diminish the educational success of students. We thus assume that greater learning difficulties under online learning conditions could affect the time in which the same level of *performance* might be achieved. Thus, greater learning difficulties might result in a study delay, especially for German students.

Hypothesis 3:
*The switch to online learning during COVID-19 has especially increased learning difficulties for German students, prolonging their study duration more in comparison to international students*.

In sum, we address how the pandemic might increase or decrease social inequalities between international and domestic students. Against the background of the Matthew effect, which claims that social inequality is rather increasing (metaphorically “the rich get richer and the poor get poorer”), we propose a more differentiated picture by addressing advantages and disadvantages for both groups during the pandemic. On the one hand, we expect an increase in social inequality due to the worsening financial burden of international students (“the poor get poorer”); on the other, we assume a decrease in social inequality due to compensational effects on the language barriers faced by international students and due to increased learning and social difficulties for German students (“the rich get poorer”). We thus argue that the pandemic has not only increased social inequalities at universities but may also have decreased particular disadvantages among international students.

## Data and Methods

### Data

We empirically test our theoretical assumptions on the basis of the nationwide German student survey “Studying in Germany in Corona Times” (Marczuk et al., 2021). This quantitative standardised survey was conducted by the German Centre for Higher Education Research and Science Studies (DZHW) and the Research Group on Higher Education at the University of Konstanz in the summer semester of 2020. The data sampling was carried out in two stages: a systematic selection of 23 universities according to their distribution across federal states and by size, subject structures and type (traditional university or university of applied sciences) was followed by a random selection of students within these institutions. The survey placed special emphasis on the challenges associated with the COVID-19 pandemic and included questions regarding the differences between the first digital semester in 2020 and the situation before the pandemic.

The total response rate was about 15%, yielding 28,623 cases. After excluding cases with missing information, we were left with an analytical sample of 20,349 valid cases. To avoid systematic bias, the data were weighted by a student's gender, university semester, field of study and type of university (for sample descriptions, see Table 5 in the Appendix). Although the data cannot be considered fully representative due to the theoretical sampling of the universities, our analytical sample shows similarities to the population of students in Germany with regard to gender and age, as well as to the subject and degree structure of the universities (Destatis, 2022).

### Variables

This study distinguishes between two groups of students: *international students* who came to Germany to study, and *German students* already living in Germany before starting university. Following official glossaries (UNESCO, 2015), we define international students as students with a non-German university entrance qualification and non-German citizenship. The data do not provide further information on migration background (i.e., country of origin, length of stay or study language), which might give further insights about the hypothesised mechanisms. International students form 7% of the sample, and this rate is lower than the rate of international students in Germany (13%) before the pandemic (Statistisches Bundesamt, 2018). This might be a result of travel restrictions to Germany during the course of COVID-19.

**Figure 1.**
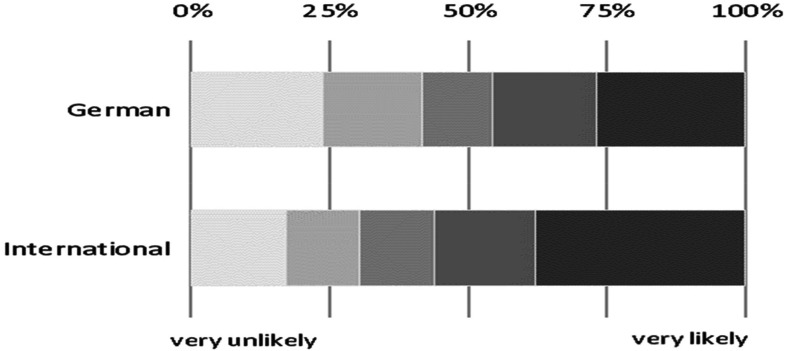
Delay in study progress by migration status (%).

*Note*. Weighted results. Source: SITCO survey 2020.

Our dependent variable addresses a prospective delay in study progress. Students could assess it by answering the question “How likely is an extension of your studies due to the Corona pandemic?” on a five-point-scale ranging from “very unlikely” to “very likely.” Although this assessment is subjective, it provides an early impression of the extent to which study progress may possibly be delayed; the real delay in study progress due to COVID-19 will only become apparent a few years from now, when students actually finish their studies. As can be seen from Figure 1, around 50% of both international and German students expect a delay in their study progress. However, this more often applies to international (AM=56%) than to German students (AM=46%, p < 0.001).

In order to explain these differences, we address possible effects of the financial situation (H1), social isolation (H2) and learning situation (H3) on the study delay of German and international students. We use items comparing these relevant aspects before and after the outbreak of the pandemic. This form of subjective comparison made by students (before and after) is more reliable for testing our assumptions about the effect of the pandemic, rather than measuring these aspects only for the current situation. All considered items are illustrated in Table 3 in the Appendix.

The *financial situation* of students is covered by two aspects: the change in students’ employment situation and the change in their parents’ financial situation. To measure the latter aspect, we consider two items. First, students were asked on a five-point-scale to what extent the income situation of their parents has worsened or improved during the pandemic. Second, students were asked whether financial support from their parents is currently limited (on a scale from 1 “does not apply at all” to 5 “fully applies”). Table 1 indicates that international students report more often than German students that the income situation of their parents has worsened during COVID-19 (.58 vs .30, p < 0.001) and that their financial support is currently limited (.50 vs .32, p < 0.001). To a lower extent, both groups also differ with regard to their employment situation: international students lost their job during the pandemic more often than German students (.27 vs .23, p < 0.01). This is in line with our theoretical assumption about the more difficult financial situation of international students during COVID-19 (H1).

**Table 1.** Descriptive Statistics Indicating Differences Between German and International Students.

	German students	International students	t-test
**Financial situation**			
Parents’ financial situation got worse	0.30	0.58	***
Financial support by parents is limited	0.32	0.50	***
I lost my student job	0.23	0.27	**
**Social isolation**			
Contact with students got more difficult	0.87	0.81	***
Communication with lecturers got more difficult	0.61	0.63	n.s.
Participating in study groups got more difficult	0.77	0.76	*
**Learning situation**			
Following course content got more difficult	0.59	0.55	***
Coping with learning material got more difficult	0.64	0.53	***
Mastering exam requirements got more difficult	0.58	0.54	***
**Observations**	24,040	1,686	25,726

Weighted frequencies. * p < 0.05. ** p < .01. *** p < .001.

All frequencies represent students reporting 4 or 5 on the five-point scale of the considered items, except for the student's job loss (yes vs no). For scale details, see Appendix Table 3.

*Source:* SITCO survey 2020.

The *social (isolation) situation* of students is measured with three items: students had to rate on a five-point-scale whether their contact with other students, their participation in study groups and their communication with lecturers has become easier or more difficult due to the pandemic. In general, the majority of students evaluate their social situation during the pandemic as more difficult (see Table 1). However, German students in particular evaluate their contact with other students (.87 vs .81, p < 0.001) and their participation in study groups (.77 vs .76, p < 0.05) as more difficult, while their communication with lecturers is not different than that reported by international students. These findings are in line with our theoretical assumptions (H2) about greater levels of social isolation experienced by German students. However, these differences are rather small compared to the financial aspects discussed above.

The *learning situation* is captured by three aspects. Students were asked whether following course content, coping with learning material and mastering exam requirements changed during the pandemic (on a scale from 1 “became easier” to 5 “became more difficult”). Most students perceive the learning situation as more difficult than before the pandemic (see Table 1). However, this more often applies to German students: following course content (.59 vs .55, p < 0.001), mastering exam requirements (.58 vs .54, p < 0.001) and especially coping with learning material (.64 vs .53, p < 0.001) seems currently to be more difficult for German than for international students. This is in line with our theoretical assumption (H3) about greater learning difficulties for German students.

## Methods

The descriptive results mainly correspond to our theoretical assumptions: international students have experienced more financial difficulties during the pandemic, while German students have reported greater difficulties with learning and social isolation. In order to test whether these components correlate with migration-specific differences in study delay, we present regression models. In particular, we run random intercept multilevel linear models (Snijders & Bosker, 2011) with universities on the higher level due to the theoretical sampling of universities in the data. In order to test the underlying mechanisms, we introduce the explanatory variables discussed above (financial, social and learning situation) into the regression models step by step. By doing so, we address which factors lead to migration-specific differences in study delay. To reduce the risk of obtaining biased results, all our models are controlled for students’ gender, age, study semester, the pursued degree and parents’ education, which are the main factors associated with educational success (for an overview, see Behr et al., 2020). For details on the control variables, see Table 5 in the Appendix.

## Results

This section presents the empirical results of the multilevel linear regression models explaining migration-specific differences in study delay (see Table 2). The models show that international students are significantly more likely to perceive a delay in study progress, even after controlling for several individual characteristics (M2 in Table 2). In the following section, we introduce distinct aspects of financial situation (M3), social isolation (M4) and learning situation (M5) in order to observe how they change the migration-specific differences in study delay.

**Table 2.** Multilevel Models of Correlations Between Changes of the Pandemic and a Delay in Study Progress.

	(M1)	(M2)	(M3)	(M4)	(M5)	(M6)
						
International students		**0** **.** 24***	0.06	**0**.26***	**0**.33***	**0**.19***
(ref. German students)		(0.05)	(0.05)	(0.05)	(0.04)	(0.04)
**Financial situation**						
Parents’ financial			**0**.18***			**0**.13***
situation got worse			(0.02)			(0.02)
Financial support by			**0**.13***			**0**.10***
parents is limited			(0.01)			(0.01)
I lost my student job			**0**.29***			**0**.22***
			(0.03)			(0.02)
**Social isolation**						
Contact with students				**0**.07***		0.00
got more difficult				(0.02)		(0.01)
Communication with				**0**.15***		0.02
lecturers got more difficult				(0.01)		(0.01)
Participating in study				**0**.16***		**0**.07***
groups got more difficult				(0.01)		(0.01)
**Learning situation**						
Following course content					**0**.13***	**0**.11***
got more difficult					(0.01)	(0.01)
Coping with learning					**0**.14***	**0**.13***
material got more difficult					(0.01)	(0.01)
Mastering exam requirements					**0**.34***	**0**.30***
got more difficult					(0.01)	(0.01)
						
Intercept	3.02	2.88	3.22	3.61	4.23	4.48
HEI level (intercept)	0.22	0.25	0.23	0.22	0.24	0.22
Individual level (residual)	1.52	1.51	1.48	1.49	1.41	1.39
AIC	74913	74675	73839	73941	71929	71348
BIC	74937	74794	73982	74084	72071	71538
Observations	20,349	20,349	20,349	20,349	20,349	20,349
ICC	0.02	0.03	0.02	0.02	0.03	0.02

* p < 0.05. ** p < .01. *** p < .001. Standard errors in parentheses.

Random intercept multilevel linear models. We use the original Likert scale (1–5) for all items regarding the financial, social or learning situation, except for the students’ job loss (yes vs no): see Table 3 in the Appendix. M2-M6 controlled for parents’ education, target degree, semester, gender, age (for full models, see Appendix Table 8). Source: SITCO survey 2020.

### Financial Situation

Model 3 (Table 2) indicates that the financial difficulties experienced by parents had a positive and highly significant effect on students’ perceived delay in study progress. Parents’ worsening income situation during COVID-19 (.18, p < 0.001) and their reduced support opportunities (.13, p < 0.001) are positively correlated with a prospective delay in the study progress of their children. The same applies to students’ job loss (.29, p < 0.001). These aspects also explain the migration-specific differences when comparing the coefficients of international students (in reference to German students): while in Model 2 we initially observed a highly significant difference between both student groups, the coefficient decreased to .06 and is no longer significant after controlling for parents’ financial situation and students’ job loss in Model 3. In line with Hypothesis 1, the more difficult financial situation of international students during the pandemic seems to make a delay in study progress more likely when compared to German students. However, additional analyses show that this is more strongly provoked by the financial situation of their parents than by their own job loss (see M3-M5 in Table 4 in the Appendix). The results thus indicate that the greater delay reported by international students compared to German ones is due to the more difficult financial situation of the former's parents living and working outside of Germany.

### Social Isolation

Besides a challenging financial situation, a higher degree of social isolation is also associated with a delay in study progress (M4 in Table 2). In particular, the more difficult the exchange in learning groups during the digital semester, the more often students perceive a delay in their study progress (.16, p < 0.001). The same applies to more challenging communication with lecturers (.15, p < 0.001) and reduced contact with other students (.07, p < 0.001). However, these aspects did not seem to affect the migration-specific differences in study delay: the coefficient only slightly increases (from .24 to .26) when considering the changed social situation (M2 vs M4). This slight increase indicates that German students worry more often about a possible study delay due to higher social isolation (suppressor effect). However, with regard to the rather small changes, we conclude that our empirical results do not support Hypothesis 2, which expected a longer study duration for German students as a result of social isolation.

### Learning Situation

In Model 5, we introduce three different aspects of the changed learning situation. In general, greater difficulty in exam preparation during the summer semester of 2020 is positively correlated with a possible study delay (.34, p < 0.001). The same applies to a more difficult learning situation with regard to following course content (.13, p < 0.001) or coping with learning material (.14,p < 0.001). With regard to the migration-specific differences, we find support for Hypothesis 3. In particular, German students suffer more than international students as a result of the changed learning situation: compared to Model 2, the coefficient of international students (with reference to German students) increases from .24 to .33 when controlling for the changed learning situation in Model 5 (suppressor effect). This means that the disadvantage of international students regarding a possible study delay would be even higher if the learning situation had not become more difficult for German students. In other words, German students struggle more with the learning situation of online modalities, and this greater difficulty is more often associated with a study delay.

In the last step of the regression analysis, we test all predictors simultaneously (M6). The migration-specific differences in study delay remain significant, but the coefficient is lower than in Model 2 (.19, p < 0.001). This is due to the two opposing processes that seem to balance each other out: although the more difficult financial situation causes a higher level of fear of a study delay for international students, German students anticipate a study delay due to a more challenging learning situation under conditions of online learning. Model 6 also reveals that the coefficients of the main predictors (the financial, social and learning situations) are relatively stable when considered all at once. However, this does not apply for the coefficients of social isolation being predominantly not significant in the overall model (M6). Additional analyses show that this is due to correlations with the learning situation. Apparently, social isolation provoked by online teaching is connected to the learning difficulties experienced by students.

### Sensitivity Checks

Our additional analyses also provide further information about the underlying mechanisms (see Table 7 in the Appendix). First, we assumed that a precarious financial situation causes higher costs (time and stress) and might distract from efficient study, thus prolonging study duration (H1). We do not have information on the time budget of students, but can consider their current stress levels. The results confirm our assumption: the positive correlation between financial situation and study delay can be partly explained by the higher stress levels reported by students (M3 vs M4 in Table 7 in the Appendix). Second, we initially expected that online teaching modalities lead to higher learning burdens due to lower levels of interaction during seminars, but at the same time can compensate for language barriers, especially when using uploaded videos (H3). We do not find support for the latter assumption: uploaded videos do not change the correlation between learning situation and study delay (M7 vs M8 in Table 7 in the Appendix). However, as already shown in the overall model, the more difficult learning situation is strongly associated with greater social isolation among students (M5-M7 in Table 7 in the Appendix). This indicates that a lower level of social interaction in online seminars does increase the learning difficulties of students, leading to a study delay.

The presented coefficients in the findings section are also robust when running additional sensitivity checks: simple linear regression models show almost identical results, indicating no bias due to the university sample of the data (see Table 6 in the Appendix). This is also represented by the low intraclass correlation coefficient showing low variance at the university level (2%–3%; see ICC Table 2). Our multilevel models also reveal that, of the three mechanisms considered, the learning difficulties most significantly explain the variance of the study delay (see AIC and BIC in M5 in Table 2). Thus, study delay is strongly associated with the differences produced by online learning conditions.

## Discussion

This paper addresses the effect of COVID-19 on the study delay of international students, a clearly disadvantaged group in German higher education. We assumed that the pandemic might have changed existing inequalities between international and domestic students. Our results show that a significant proportion of both groups expect a study delay due to COVID-19. However, this more often applies to international students. This disadvantage of international students is fully explained by the more difficult financial situation of their parents. Apparently, the financial situation of parents working abroad is more negatively affected by the pandemic compared to German parents. Their support is therefore limited, resulting in higher stress levels of international students, distracting them from efficient studying. However, at the same time our results reveal increased burdens for German students: they more often expect a delay in study progress due to experiencing greater difficulties with online learning. There are indications that German students miss out on interacting and discussing with fellow students due to digital formats, and that they thus struggle more with learning. This emphasises that online teaching might generate more difficulties in particular countries: since learning in Germany heavily relies on discussion and interaction (Heublein, 2015), the German discursive teaching tradition might be more difficult to maintain when teaching switches from in-person to online formats.

This study proposes a differentiated picture of the impact of COVID-19 on social inequality. Financially poor international students have got poorer, but more advantaged German students have also missed out by struggling more with online learning. This differentiated picture not only emphasises the increased difficulties of disadvantaged groups (as pointed out by previous research), but also sheds light on the struggles of the advantaged group.

Several recommendations for (online) teaching and support structures at the host universities arise from our findings. They emphasise the need for more social interaction in online seminars: introducing innovative methods, such as collaborative learning methods, might further increase students’ cooperation. Using methods of design thinking (DT) —such as affinity diagrams, empathy maps and user stories—might in turn increase the creativity of students when co-working on assignments (Hochschulforum_Digitalisierung, 2021). These interactive aspects might be especially important for the German discursive context, which heavily relies on these kinds of interactions. Universities should see the changed study situation as an opportunity to anchor virtual mobility in the curriculum and offer virtual teaching programs. As Lee et al. (2022) assume, this could be a way to increase the number of international students. In addition, domestic students profit from collaborative online international learning (COIL) tools with regard to intercultural competencies (De Hei et al., 2020).

In addition, our results indicate a clear need for more targeted financial support: student groups clearly affected by the pandemic should be identified and better supported. While Germany has introduced several new COVID-19-related loans or allowed an extension on federal loans (BAföG), these measures have been aimed at German students, leaving international students behind. Although providing financial support generally lies outside the duties of universities, they could provide information on financial aspects and job regulations. These and other student services should be visible and proactive in approaching international students in particular. Making information available in other languages apart from German could further diminish the information gap between international and German students.

Despite these contributions and recommendations, our study reveals some limitations. The causality assumptions are limited by the cross-sectional data, and because of the subjective self-reporting questionnaire, social desirability effects could bias the results. Another limitation is that we cannot consider the heterogeneous group of international students with regard to their country of origin, which might have a different impact on the main mechanisms. For instance, the financial situation of international students from countries with greater social welfare support (Sweden, Norway, the Netherlands, etc.) might be less problematic than assumed in the paper, and the language barrier should not apply to German-speaking international students from Austria or Switzerland or to English-speaking students studying in programs delivered in English at German universities.

Future research should thus consider these aspects by acquiring data that can provide more in-depth information on international students (country of origin, language, length of stay). In order to gain a deeper understanding of the changed situation of international students, it would be advisable to use qualitative methods in addition to quantitative research approaches (mixed methods approach). From an intersectional perspective, it would be important to know if international students with specific characteristics (gender, parental education, etc.) have particular difficulties in higher education. Future research should also track current and future outcomes during the pandemic: while we considered the study situation immediately after its outbreak, it remains unclear if this situation got better or worse over the following semesters. Also, further analyses using longitudinal data should allow tracing the actual study duration and show the extent to which the pandemic indeed increased it. Finally, considering the impact of COVID-19 from an international perspective might give further insights into country-specific struggles provoked by the pandemic. In this regard, it would be important to learn which education systems managed the COVID-19 crisis better or worse.

## Supplemental Material

sj-docx-1-jsi-10.1177_10283153221150116 - Supplemental material for Did the Poor Get Poorer? The Impact of COVID-19 on Social Inequalities Between International and Domestic Students Click here for additional data file. Supplemental material, sj-docx-1-jsi-10.1177_10283153221150116 for Did the Poor Get Poorer? The Impact of COVID-19 on Social Inequalities Between International and Domestic Students by Anna Marczuk and Markus Lörz in Journal of Studies in International Education
