# Supplementary material for: Did the Poor Get Poorer? The Impact of COVID-19 on Social Inequalities Between International and Domestic Students
Source: J Stud Int Educ. 2023 Jan 11:10283153221150116. doi: 10.1177/10283153221150116 (PMC9843152; doi:10.1177/10283153221150116)
Supplement: sj-docx-1-jsi-10.1177_10283153221150116 - Supplemental material for Did the Poor Get Poorer? The Impact of COVID-19 on Social Inequalities Between International and Domestic Students [file sj-docx-1-jsi-10.1177_10283153221150116.docx]

**Appendix**

**Table 3: Measurements of the main variables**

|  | Measurement |
| --- | --- |
| **Study delay** | *How likely is an extension of your studies due to the Corona pandemic?*  1 “very unlikely” – 5 “very likely” |
|  | *How have the following aspects changed for you as a result of the Corona pandemic?*  1 “became easier” – 5 “became more difficult” |
| **Learning situation** | - Following course content |
|  | - Coping with learning material |
|  | - Mastering exam requirements |
| **Social isolation** | - Contact with students |
|  | - Communication with lecturers |
|  | - Participating in study groups |
| **Financial situation** | *Has the Corona pandemic affected your parents' income situation?*  The income situation has... 1 “became easier” – 5 “became more difficult” |
|  | *To what extent do the following statements apply to you and your financial situation?*  My parents are only limited in their ability to support me financially.  1 “does not apply at all” – 5 “fully applies” |
|  | *Has the Corona pandemic affected your employment?* Yes, I was laid off (yes/no)  (No = students answering “no” or those not employed before the pandemic) |

Source: SITCO survey 2020

**Table 4: Multilevel models of correlations between changes of the pandemic and a delay in study progress (expanded Table 2)**

|  | (M1) | (M2) | (M3) | (M4) | (M5) | (M6) | (M7) | (M8) |
| --- | --- | --- | --- | --- | --- | --- | --- | --- |
|  |  |  |  |  |  |  |  |  |
| International students |  | **0.22^***^** | **0.24^***^** | 0.06 | **0.22^***^** | **0.26^***^** | **0.33^***^** | **0.19^***^** |
| (ref. German students) |  | (0.04) | (0.05) | (0.05) | (0.05) | (0.05) | (0.04) | (0.04) |
| **Financial situation** |  |  |  |  |  |  |  |  |
| Parents’ financial |  |  |  | **0.21^***^** |  |  |  | **0.13^***^** |
| situation got worse |  |  |  | (0.02) |  |  |  | (0.02) |
| Financial support by |  |  |  | **0.14^***^** |  |  |  | **0.10^***^** |
| parents is limited |  |  |  | (0.01) |  |  |  | (0.01) |
| I lost my student job |  |  |  |  | **0.40^***^** |  |  | **0.22^***^** |
|  |  |  |  |  | (0.02) |  |  | (0.02) |
| **Social isolation** |  |  |  |  |  |  |  |  |
| Contact with students |  |  |  |  |  | **0.07^***^** |  | 0.00 |
| got more difficult |  |  |  |  |  | (0.02) |  | (0.01) |
| Communication with |  |  |  |  |  | **0.15^***^** |  | 0.02 |
| lecturers got more difficult |  |  |  |  |  | (0.01) |  | (0.01) |
| Participating in study |  |  |  |  |  | **0.16^***^** |  | **0.07^***^** |
| groups got more difficult |  |  |  |  |  | (0.01) |  | (0.01) |
| **Learning situation** |  |  |  |  |  |  |  |  |
| Following course content |  |  |  |  |  |  | **0.13^***^** | **0.11^***^** |
| got more difficult |  |  |  |  |  |  | (0.01) | (0.01) |
| Coping with learning |  |  |  |  |  |  | **0.14^***^** | **0.13^***^** |
| material got more difficult |  |  |  |  |  |  | (0.01) | (0.01) |
| Mastering exam requirements |  |  |  |  |  |  | **0.34^***^** | **0.30^***^** |
| got more difficult |  |  |  |  |  |  | (0.01) | (0.01) |
|  |  |  |  |  |  |  |  |  |
| **Control variables** |  |  |  |  |  |  |  |  |
| First-generation students |  |  | **0.11^***^** | -0.03 | **0.10^***^** | **0.13^***^** | **0.11^***^** | 0.00 |
|  |  |  | (0.02) | (0.02) | (0.02) | (0.02) | (0.02) | (0.02) |
| *Target degree (ref. BA)* |  |  |  |  |  |  |  |  |
| MA |  |  | **-0.14^***^** | **-0.12^***^** | **-0.12^***^** | **-0.14^***^** | **-0.13^***^** | **-0.11^***^** |
|  |  |  | (0.03) | (0.03) | (0.03) | (0.03) | (0.03) | (0.03) |
| State examination |  |  | **-0.28^***^** | **-0.26^***^** | **-0.27^***^** | **-0.27^***^** | **-0.30^***^** | **-0.28^***^** |
|  |  |  | (0.03) | (0.03) | (0.03) | (0.03) | (0.03) | (0.03) |
| Diploma |  |  | **0.45^**^** | **0.47^***^** | **0.45^**^** | **0.40^**^** | **0.44^***^** | **0.44^***^** |
|  |  |  | (0.14) | (0.14) | (0.14) | (0.14) | (0.13) | (0.13) |
| Other |  |  | **-0.27^*^** | **-0.28^*^** | **-0.25^+^** | -0.21 | -0.19 | -0.18 |
|  |  |  | (0.14) | (0.13) | (0.14) | (0.13) | (0.13) | (0.13) |
| *Gender (ref. male)* |  |  |  |  |  |  |  |  |
| Female |  |  | **-0.17^***^** | **-0.18^***^** | **-0.18^***^** | **-0.17^***^** | **-0.23^***^** | **-0.24^***^** |
|  |  |  | (0.02) | (0.02) | (0.02) | (0.02) | (0.02) | (0.02) |
| Diverse |  |  | **0.54^***^** | **0.42^***^** | **0.52^***^** | **0.48^***^** | **0.32^**^** | **0.23^*^** |
|  |  |  | (0.12) | (0.12) | (0.12) | (0.12) | (0.11) | (0.11) |
| Age |  |  | **0.01^**^** | -0.00 | **0.01^**^** | **0.01^**^** | **0.00^*^** | -0.00 |
|  |  |  | (0.00) | (0.00) | (0.00) | (0.00) | (0.00) | (0.00) |
| Semester |  |  | **0.02^**^** | **0.02^*^** | **0.02^*^** | **0.02^**^** | **0.05^***^** | **0.04^***^** |
|  |  |  | (0.01) | (0.01) | (0.01) | (0.01) | (0.01) | (0.01) |
|  |  |  |  |  |  |  |  |  |
| Intercept | 3.02 | 3.01 | 2.88 | 3.32 | 2.82 | 3.61 | 4.23 | 4.48 |
| HEI level (intercept) | 0.22 | 0.22 | 0.25 | 0.24 | 0.24 | 0.22 | 0.24 | 0.22 |
| Individual level (residual) | 1.52 | 1.52 | 1.51 | 1.49 | 1.50 | 1.49 | 1.41 | 1.39 |
| Observations | 20349 | 20349 | 20349 | 20349 | 20349 | 20349 | 20349 | 20349 |
| ICC | 0.02 | 0.02 | 0.03 | 0.03 | 0.02 | 0.02 | 0.03 | 0.02 |

* p < 0.05, ** p < .01, *** p < .001. Standard errors in parentheses. We use the original Likert scale (1–5) for all items regarding the financial, social or learning situation, except for the students’ job loss (yes vs no): see Table 3. Source: SITCO survey 2020

**Table 5: Sample descriptions**

| **Items** | **Mean** | **SD** | **Min.** | **Max.** |
| --- | --- | --- | --- | --- |
| Study delay due to COVID-19^1^ | 3.12 | 1.54 | 1 | 5 |
| International students | 0.07 |  | 0 | 1 |
| First-generation students | 0.45 |  | 0 | 1 |
| *Gender* |  |  |  |  |
| Male | 0.38 |  | 0 | 1 |
| Female | 0.60 |  | 0 | 1 |
| Diverse | 0.02 |  | 0 | 1 |
| Age | 24.36 | 4.61 | 17 | 45 |
| *Target degree* |  |  |  |  |
| BA | 0.57 |  | 0 | 1 |
| MA | 0.25 |  | 0 | 1 |
| State examination | 0.15 |  | 0 | 1 |
| Diploma | 0.01 |  | 0 | 1 |
| Other | 0.02 |  | 0 | 1 |
| Study semester | 3.32 | 1.97 | 1 | 8 |
| *Learning situation changed due to COVID-19^2^* |  |  |  |  |
| Following course content | 3.68 | 1.21 | 1 | 5 |
| Coping with learning material | 3.89 | 1.14 | 1 | 5 |
| Mastering exam requirements | 3.76 | 0.97 | 1 | 5 |
| *Social situation changed due to COVID-19^2^* |  |  |  |  |
| Contact with students | 4.49 | 0.81 | 1 | 5 |
| Communication with lecturers | 3.76 | 0.99 | 1 | 5 |
| Participating in study groups | 4.19 | 0.96 | 1 | 5 |
| *Financial situation changed* |  |  |  |  |
| Parents’ financial situation changed due to  COVID-19^2^ | 3.39 | 0.68 | 1 | 5 |
| Financial support by parents is limited^3^ | 2.58 | 1.60 | 1 | 5 |
| I lost my student job | 0.24 |  | 0 | 1 |
| Digital teaching: Courses with uploaded videos^4^ | 2.60 | 1.17 | 1 | 5 |
| *Current psychological burden^5^* |  |  |  |  |
| I am feeling stressed | 3.85 | 1.07 | 1 | 5 |
| I am feeling overloaded | 3.58 | 1.16 | 1 | 5 |
| I am feeling gloomy | 2.67 | 1.08 | 1 | 5 |
| I am feeling depressed | 2.74 | 1.09 | 1 | 5 |
| Observations | 19,814 |  |  |  |

^1^ Scale: 1 ”very unlikely” – 5 “very likely”

^2^ Scale: 1 “became easier” – 5 “became more difficult”

^3^ Scale: 1 “does not apply at all” – 5 “fully applies”

^4^ Scale: 1 “none” – 5 “all”

^5^ Scale: 1 “not at all” – 5 “very strong”

Source: SITCO survey 2020

**Table 6: OLS models of correlations between changes of the pandemic and a delay in study progress**

|  | (M1) | (M2) | (M3) | (M4) | (M5) | (M6) | (M7) |
| --- | --- | --- | --- | --- | --- | --- | --- |
| International students | **0.28^***^** | **0.30^***^** | **0.11^*^** | **0.28^***^** | **0.32^***^** | **0.39^***^** | **0.24^***^** |
| (ref. German students) | (0.05) | (0.05) | (0.05) | (0.05) | (0.05) | (0.04) | (0.04) |
| **Financial situation** |  |  |  |  |  |  |  |
| Parents’ financial |  |  | **0.21^***^** |  |  |  | **0.13^***^** |
| situation got worse |  |  | (0.02) |  |  |  | (0.02) |
| Financial support by |  |  | **0.15^***^** |  |  |  | **0.11^***^** |
| parents is limited |  |  | (0.01) |  |  |  | (0.01) |
| I lost my student job |  |  |  | **0.42^***^** |  |  | **0.24^***^** |
|  |  |  |  | (0.03) |  |  | (0.02) |
| **Social isolation** |  |  |  |  |  |  |  |
| Contact with students |  |  |  |  | **0.09^***^** |  | **0.03^*^** |
| got more difficult |  |  |  |  | (0.02) |  | (0.01) |
| Communication with |  |  |  |  | **0.15^***^** |  | 0.02 |
| lecturers got more difficult |  |  |  |  | (0.01) |  | (0.01) |
| Participating in study |  |  |  |  | **0.17^***^** |  | **0.08^***^** |
| groups got more difficult |  |  |  |  | (0.01) |  | (0.01) |
| **Learning situation** |  |  |  |  |  |  |  |
| Following course content |  |  |  |  |  | **0.12^***^** | **0.09^***^** |
| got more difficult |  |  |  |  |  | (0.01) | (0.01) |
| Coping with learning |  |  |  |  |  | **0.15^***^** | **0.13^***^** |
| material got more difficult |  |  |  |  |  | (0.01) | (0.01) |
| Mastering exam requirements |  |  |  |  |  | **0.34^***^** | **0.30^***^** |
| got more difficult |  |  |  |  |  | (0.01) | (0.01) |
| Intercept | 3.10 | 3.01 | 3.45 | 2.94 | 3.74 | 4.35 | 4.61 |
| Observations | 20,349 | 20,349 | 20,349 | 20,349 | 20,349 | 20,349 | 20,349 |
| *R*^2^ | 0.002 | 0.013 | 0.049 | 0.026 | 0.052 | 0.134 | 0.162 |

* p < 0.05, ** p < .01, *** p < .001. Standard errors in parentheses.

We use the original Likert scale (1–5) for all items regarding the financial, social or learning situation, except for the students’ job loss (yes vs no): see Table 3.

Models 2–7 controlled for educational background, gender, age, target degree and semester.

Source: SITCO survey 2020

**Table 7: Correlations of mechanism explaining the delay in study progress**

|  | (M1) | (M2) | (M3) | (M4) | (M5) | (M6) | (M7) | (M8) | (M9) |
| --- | --- | --- | --- | --- | --- | --- | --- | --- | --- |
|  |  |  |  |  |  |  |  |  |  |
| International students (Ref. German students) |  | **0.24^***^** | 0.06 | 0.06 | **0.26^***^** | **0.33^***^** | **0.33^***^** | **0.33^***^** | **0.16^***^** |
| **Financial situation** |  |  |  |  |  |  |  |  |  |
| Parents’ financial situation got worse |  |  | **0.19^***^** | **0.14^***^** |  |  |  |  | **0.11^***^** |
| Financial support by parents is limited |  |  | **0.13^***^** | **0.09^***^** |  |  |  |  | **0.09^***^** |
| I lost my student job |  |  | **0.29^***^** | **0.26^***^** |  |  |  |  | **0.21^***^** |
| **Psychological burden^1^** |  |  |  |  |  |  |  |  |  |
| I am stressed |  |  |  | **0.11^***^** |  |  |  |  | **0.05^***^** |
| I am overloaded |  |  |  | **0.18^***^** |  |  |  |  | **0.08^***^** |
| I feel gloomy |  |  |  | **0.07^***^** |  |  |  |  | **0.07^***^** |
| I feel depressed |  |  |  | **0.10^***^** |  |  |  |  | **0.07^***^** |
| **Social isolation** |  |  |  |  |  |  |  |  |  |
| Contact with students got more difficult |  |  |  |  | **0.06^***^** | 0.00 |  |  | 0.00 |
| Communication with lecturers got more difficult |  |  |  |  | **0.15^***^** | 0.02 |  |  | 0.01 |
| Participating in study groups got more difficult |  |  |  |  | **0.17^***^** | **0.08^***^** |  |  | **0.07^***^** |
| **Learning situation** |  |  |  |  |  |  |  |  |  |
| Following course content got more difficult |  |  |  |  |  | **0.12^***^** | **0.13^***^** | **0.13^***^** | **0.10^***^** |
| Coping with learning material got more difficult |  |  |  |  |  | **0.14^***^** | **0.14^***^** | **0.14^***^** | **0.09^***^** |
| Mastering exam requirements got more difficult |  |  |  |  |  | **0.32^***^** | **0.33^***^** | **0.33^***^** | **0.26^***^** |
| **Digital teaching: uploaded videos^2^** |  |  |  |  |  |  |  | **-0.02^*^** | -0.01 |
| Intercept | 3.02 | 2.88 | 3.33 | 1.74 | 3.60 | 4.35 | 4.23 | 4.29 | 3.43 |
| HEI level (intercept) | 0.23 | 0.25 | 0.23 | 0.21 | 0.22 | 0.23 | 0.24 | 0.24 | 0.21 |
| Individual level (residual) | 1.52 | 1.51 | 1.48 | 1.43 | 1.48 | 1.41 | 1.41 | 1.41 | 1.38 |
| Observations | 19,814 | 19,814 | 19,814 | 19,814 | 19,814 | 19,814 | 19,814 | 19,814 | 19,814 |
| ICC | 0.02 | 0.03 | 0.02 | 0.02 | 0.02 | 0.03 | 0.03 | 0.03 | 0.02 |

* p < 0.05, ** p < .01, *** p < .001.

We use the original Likert scale (1–5) for all items regarding the financial, social or learning situation, except for the students’ job loss (yes vs no): see Table 3.

^1^ *How have you felt for the most part in the last four weeks?* Scale: 1 “not at all” – 5 “very strong”

^2^ *How many of your courses are organized this semester exclusively as…uploaded videos?* Scale: 1 “none” - 5 “all”

Models 2–9 controlled for educational background, gender, age, target degree and semester.

Source: SITCO survey 2020

**Table 8: Multilevel models of correlations between changes of the pandemic and a delay in study progress (Table 2 including control variables)**

|  | (M1) | (M2) | (M3) | (M4) | (M5) | (M6) |
| --- | --- | --- | --- | --- | --- | --- |
|  |  |  |  |  |  |  |
| International students |  | **0.24^***^** | 0.06 | **0.26^***^** | **0.33^***^** | **0.19^***^** |
| (ref. German students) |  | (0.05) | (0.05) | (0.05) | (0.04) | (0.04) |
| **Financial situation** |  |  |  |  |  |  |
| Parents’ financial |  |  | **0.18^***^** |  |  | **0.13^***^** |
| situation got worse |  |  | (0.02) |  |  | (0.02) |
| Financial support by |  |  | **0.13^***^** |  |  | **0.10^***^** |
| parents is limited |  |  | (0.01) |  |  | (0.01) |
| I lost my student job |  |  | **0.29^***^** |  |  | **0.22^***^** |
|  |  |  | (0.03) |  |  | (0.02) |
| **Social isolation** |  |  |  |  |  |  |
| Contact with students |  |  |  | **0.07^***^** |  | 0.00 |
| got more difficult |  |  |  | (0.02) |  | (0.01) |
| Communication with |  |  |  | **0.15^***^** |  | 0.02 |
| lecturers got more difficult |  |  |  | (0.01) |  | (0.01) |
| Participating in study |  |  |  | **0.16^***^** |  | **0.07^***^** |
| groups got more difficult |  |  |  | (0.01) |  | (0.01) |
| **Learning situation** |  |  |  |  |  |  |
| Following course content |  |  |  |  | **0.13^***^** | **0.11^***^** |
| got more difficult |  |  |  |  | (0.01) | (0.01) |
| Coping with learning |  |  |  |  | **0.14^***^** | **0.13^***^** |
| material got more difficult |  |  |  |  | (0.01) | (0.01) |
| Mastering exam requirements |  |  |  |  | **0.34^***^** | **0.30^***^** |
| got more difficult |  |  |  |  | (0.01) | (0.01) |
|  |  |  |  |  |  |  |
| **Control variables** |  |  |  |  |  |  |
| First-generation students |  | **0.11^***^** | -0.03 | **0.13^***^** | **0.11^***^** | 0.00 |
|  |  | (0.02) | (0.02) | (0.02) | (0.02) | (0.02) |
| *Target degree (ref. BA)* |  |  |  |  |  |  |
| MA |  | **-0.14^***^** | **-0.10^***^** | **-0.14^***^** | **-0.13^***^** | **-0.11^***^** |
|  |  | (0.03) | (0.03) | (0.03) | (0.03) | (0.03) |
| State examination |  | **-0.28^***^** | **-0.25^***^** | **-0.27^***^** | **-0.30^***^** | **-0.28^***^** |
|  |  | (0.03) | (0.03) | (0.03) | (0.03) | (0.03) |
| Diploma |  | **0.45^**^** | **0.47^***^** | **0.40^**^** | **0.44^***^** | **0.44^***^** |
|  |  | (0.14) | (0.14) | (0.14) | (0.13) | (0.13) |
| Other |  | **-0.27^*^** | **-0.26^*^** | -0.21 | -0.19 | -0.18 |
|  |  | (0.14) | (0.13) | (0.13) | (0.13) | (0.13) |
| *Gender (ref. male)* |  |  |  |  |  |  |
| Female |  | **-0.17^***^** | **-0.19^***^** | **-0.17^***^** | **-0.23^***^** | **-0.24^***^** |
|  |  | (0.02) | (0.02) | (0.02) | (0.02) | (0.02) |
| Diverse |  | **0.54^***^** | **0.41^***^** | **0.48^***^** | **0.32^**^** | **0.23^*^** |
|  |  | (0.12) | (0.12) | (0.12) | (0.11) | (0.11) |
| Age |  | **0.01^**^** | 0.00 | **0.01^**^** | **0.00^*^** | -0.00 |
|  |  | (0.00) | (0.00) | (0.00) | (0.00) | (0.00) |
| Semester |  | **0.02^**^** | **0.01^*^** | **0.02^**^** | **0.05^***^** | **0.04^***^** |
|  |  | (0.01) | (0.01) | (0.01) | (0.01) | (0.01) |
|  |  |  |  |  |  |  |
| Intercept | 3.02 | 2.88 | 3.22 | 3.61 | 4.23 | 4.48 |
| HEI level (intercept) | 0.22 | 0.25 | 0.23 | 0.22 | 0.24 | 0.22 |
| Individual level (residual) | 1.52 | 1.51 | 1.48 | 1.49 | 1.41 | 1.39 |
| Observations | 20,349 | 20,349 | 20,349 | 20,349 | 20,349 | 20,349 |
| ICC | 0.02 | 0.03 | 0.02 | 0.02 | 0.03 | 0.02 |

* p < 0.05, ** p < .01, *** p < .001. Standard errors in parentheses.

Random intercept multilevel linear models. We use the original Likert scale (1–5) for all items regarding the financial, social or learning situation, except for the students’ job loss (yes vs no): see Table 3. Source: SITCO survey 2020
